# Comparative Analysis of Physical Demands and Physiological Responses of Different Warm-Up Protocols in Highly Trained Female Football Players

**DOI:** 10.3390/s26103207

**Published:** 2026-05-19

**Authors:** Ibai Errekagorri, Aratz Olaizola, Julen Castellano, Eduardo Abade, Hugo Silva

**Affiliations:** 1 Department of Physical Education and Sport, Faculty of Education and Sport, University of the Basque Country (EHU), 01007 Vitoria-Gasteiz, Spain; olaizola525@gmail.com (A.O.); julen.castellano@ehu.eus (J.C.); 2 Research Center in Sports Sciences, Health Sciences and Human Development (CIDESD), University of Trás-Os-Montes e Alto Douro (UTAD), 5000-801 Vila Real, Portugal; eduardoabade@utad.pt; 3 Departament of Sports Sciences, Exercise and Health, University of Trás-Os-Montes e Alto Douro (UTAD), 5000-801 Vila Real, Portugal; 4 Portugal Football School, Portuguese Football Federation, 1495-433 Oeiras, Portugal; 5 Research Center in Sports Sciences, Health Sciences and Human Development (CIDESD), University of Maia, 4475-690 Maia, Portugal; hugorodriguessilva.35@gmail.com

**Keywords:** activation, electronic performance and tracking systems, soccer, time-motion analysis, women

## Abstract

This study aimed to describe and compare warm-up (WU) demands of different protocols (with ball, without ball and pre-match) on female football players. Twenty-two players belonging to the same team were monitored throughout 12 training weeks using global navigation satellite system technology sampling at 10 Hz. The variables used were duration, total distance, distances covered within 0–45%, 45–60%, 60–85%, and 85–100% of individual maximum speed, maximum speed, maximum acceleration, maximum deceleration, number of accelerations of magnitudes between 1 and 2 m/s^2^, 2 and 3 m/s^2^, 3 and 4 m/s^2^, and above 4 m/s^2^, number of decelerations of magnitudes between −1 and −2 m/s^2^, −2 and −3 m/s^2^, −3 and −4 m/s^2^, and below −4 m/s^2^, Player Load, and TRIMP Edwards. A linear mixed model was carried out for each variable in order to analyze the differences between WU protocols. The main results were that pre-match WU showed higher values in the following variables: (1) duration; (2) distances covered within 60–85% and 85–100% of individual maximum speed; (3) maximum speed, acceleration and deceleration; (4) number of accelerations of magnitudes between 2 and 3 m/s^2^ and between 3 and 4 m/s^2^, and more decelerations of magnitudes between −2 and −3 m/s^2^, −3 and −4 m/s^2^ and below −4 m/s^2^; and, (5) TRIMP Edwards. These findings underscore the importance of WU design in preparing female football players for high-intensity match demands and contribute to the development of specific WU strategies for them.

## 1. Introduction

Warm-up (WU) routines are purposefully structured to prepare football players both physiologically and psychologically for the demands of upcoming physical activities, whether during training sessions or competitive events [1]. In addition to elevating core body and muscle temperatures, WUs contribute to optimizing athletic performance by activating key neuromuscular functions and potentially reducing the risk of injury [1,2,3]. Research has shown that effective WU protocols can lead to measurable improvements in specific physical qualities of team sports players such as sprinting speed [4], vertical jump performance [5], change in direction ability [6], and overall agility [4]. These enhancements are crucial, as they directly influence the athlete’s readiness and effectiveness in the subsequent physical activity, whether in training or competition settings.

Before designing WUs, practitioners should consider various factors, such as duration, intensity, density or load, and whether the WU is passive or active, as these can differently influence the effectiveness of the protocols [1,7]. To maximize the effectiveness of WUs, previous research has offered specific recommendations regarding their structure and content [8]. Notably, several studies emphasize the importance of prioritizing intensity over volume to achieve performance benefits [1,4,9,10]. In this regard, the physiological and metabolic demands imposed during WU may directly influence subsequent exercise responses. Previous literature has shown that WU may enhance performance through mechanisms such as increased muscle temperature, elevated baseline oxygen consumption, enhanced anaerobic metabolism, and post-activation potentiation [2]. However, excessively demanding WU protocols may also induce excessive metabolic stress and compromise players’ readiness for the subsequent activity if intensity and duration are not appropriately managed [2]. In football, although high-intensity efforts, such as maximum sprint speed, can be achieved during portions of a traditional 23 min WU, a shorter and more targeted 12 min protocol using a 3 vs. 3 small-sided game format (three bouts of 2 min of play with 2 min rest intervals) has demonstrated greater effectiveness in enhancing countermovement jump performance and reactive agility [10]. A study provided another interesting example [11] that compared the same WU structure but with three different durations (8, 15 and 25 min), with the shorter option being the only one leading to improvements in 10- and 20 m sprint times. Additionally, the time-efficient nature of this approach creates opportunities for coaching staff to integrate learning or developmental stimuli within the WU itself, as previously suggested [12].

However, the desired intensity needs to be carefully monitored to ensure that players are indeed exposed to WUs, including high-intensity actions, without compromising the readiness of the subsequent activity due to excessive fatigue. To achieve this, sports science practitioners can collect valuable data that translates to the amount of work done by players, often referred to as external load, and include data such as total distance covered, high-speed displacements, accelerations and decelerations, among others [13]. While this monitoring procedure is commonly applied to collect data from both training sessions and competition [14,15,16], assessing activities as an overall, without distinguishing between them, may compromise the specific analysis of each individual activity. However, different WU strategies will ultimately result in different demands. For example, a previous study reported higher accelerative and decelerative demands during a WU with shuttle drills in comparison with a reaction-based WU [17]. Additionally, when comparing the relative demands (per minute) of the pre-match WU with the actual match demands, one study [18] reported similar distances covered below 12 km/h and the number of accelerations > 3 m/s^2^. This provides an interesting insight, referring to a potential similarity of intensities between WUs and the subsequent activity. That is, considering that matches are frequently the most demanding moment of the week [13], pre-match WU would be more intense than WUs that precede lower intensities training sessions. However, due to the limited available data, it is not possible to draw more definitive conclusions regarding whether different WU intensities are specifically registered during pre-matches or training sessions WU.

Additionally, there is a notable gap in research involving female athletes, prompting recent calls for increased attention to this area [8,19,20]. The scarcity of scientific evidence specific to female populations may lead practitioners to inappropriately apply findings derived from male athletes to female contexts [21]. Therefore, understanding the demands placed on players, female players, during WU strategies would contribute valuable insights into this fundamental component of football preparation for this underrepresented population. Therefore, the present study aimed to describe and compare the physical demands and physiological responses of various WU protocols in training sessions and official league matches.

## 2. Materials and Methods

### 2.1. Study Design

An observational analytical study, with a cross-sectional repeated-measures design, was conducted across 12 microcycles during an official competitive season.

### 2.2. Participants

A total of 22 semi-professional (Tier 3—highly trained) [22] female football players (age: 24.6 ± 4.0 years; height: 163.9 ± 5.0 cm; body mass: 58.5 ± 4.2 kg) belonging to the same football team of the Spanish Second Women’s Division participated in this study. It should be noted that all players included in this study were fully fit for participation throughout the monitoring period, with no injuries, medical restrictions, or need for modified training participation. Due to the particularities of the position, goalkeeper data were excluded. Playing positions included central defenders (n = 4), fullbacks (n = 4), midfielders (n = 8) and forwards (n = 6). A total of 398 individual observations were analyzed.

### 2.3. Training and Match Warm-Up Protocols

In this study three types of WU protocols were analyzed: two training sessions WUs (i.e., with ball and without ball) and one pre-match WU. It is important to note that the different WU protocols were not implemented as a continuous sequence within the same session, but rather were integrated on different days of the microcycle according to the objectives of each session.

WU with ball (n = 86 individual observations): This protocol followed a dynamic and specific approach that incorporates ball-related activities and higher-intensity drills that required decision-making. Common exercises in this phase included opposed lines with explosive starts to stimulate reaction and speed; passing wheels with movement, focusing on passing, displacement, and spatial orientation; micro-activations such as duels (e.g., 1 vs. 1 or 2 vs. 1), which combined cognitive demands and physical effort; pair passing drills over short, medium, and long distances; and small-sided possession games with technical objectives such as wall passes, support play, or switching the point of attack. This phase aimed to complete neuromuscular activation, prepare players specifically for the session’s demands, and connect body and mind through game-relevant scenarios. In most cases, this WU protocol was implemented during the middle days of the week (MD-4 and MD-3), coinciding with acquisition-oriented sessions that required specific ball-related activation.

WU without ball (n = 244 individual observations): This protocol aimed to prepare players for upcoming drills through general body activation, with a focus on joint mobility, dynamic stretching, movement skills, and fundamental motor patterns. The players then performed actions that required them to force acceleration and deceleration, in addition to some high-intensity long runs. It was typically performed in a circular formation to encourage collective engagement and facilitate a smooth transition to more specific exercises. The emphasis was on work without the ball, with the primary goal of globally activating the body, improving mobility, and preparing players both physically and mentally for the session. This warm-up protocol was mainly implemented on introductory days (MD-5) and on days close to competition (MD-1), where the objective was a more general activation.

Pre-match WU (n = 68 individual observations): This protocol was structured sequentially to achieve optimal activation before the match. It began with a free activation, involving gentle movements and spontaneous locomotion. Joint mobility exercises were then performed targeting the main joints involved in play, such as ankles, knees, hips, and shoulders. This was followed by paired passing drills to refine ball touch and coordination, followed by possession drills in tight spaces to train decision-making, tempo, and technical precision. The sequence continued with a drill of attack vs. defense, in which the intensity was gradually increased to approximate that of actual match play. Then, unopposed or semi-opposed finishing drills were conducted, focusing on technical execution and accuracy. This WU protocol concluded with short and long sprints to provide final high-speed stimuli and simulate the opening actions of the match.

### 2.4. Variables

For all WU protocols the following variables were retrieved: duration (in min), total distance, distances covered within 0–45% (D0–45), 45–60% (D45–60), 60–85% (D60–85), and 85–100% (D85–100) of individual maximum speed, maximum speed, maximum acceleration, maximum deceleration, number of accelerations of magnitudes between 1 and 2 m/s^2^ (nACC1–2), 2 and 3 m/s^2^ (nACC2–3), 3 and 4 m/s^2^ (nACC3–4) and above 4 m/s^2^ (nACC4), number of decelerations of magnitudes between −1 and −2 m/s^2^ (nDEC1–2), −2 and −3 m/s^2^ (nDEC2–3), −3 and −4 m/s^2^ (nDEC3–4) and below −4 m/s^2^ (nDEC4), Player Load, and TRIMP Edwards.

In order to obtain the relative values (%) of individual maximum speed for each player, the maximum reference value was assessed throughout a maximum 40 m sprint test. Regarding the TRIMP Edwards, it should be noted that it is a method for quantifying internal training load based on heart rate, calculated by multiplying the time spent in each heart rate zone by a corresponding weighting factor. Specifically, the time spent in 50–60% of maximum heart rate is multiplied by 1; 60–70% by 2; 70–80% by 3; 80–90% by 4; and 90–100% by 5. These zones are automatically identified and updated by the monitoring software, providing a weighted score that reflects both the intensity and duration of effort during a session [23]. However, the maximum heart rate was determined individually for each player after the completion of the 12-week monitoring period. Specifically, the highest heart rate value recorded for each player across all monitored training sessions and official matches was used as their individual maximum heart rate. This value was then used to establish the five heart rate zones required for the Edwards’ TRIMP calculation. Finally, it should be noted that, except for maximum values (i.e., speed, acceleration and deceleration), all variables were expressed per minute.

### 2.5. Procedures

The players were monitored with WIMU^®^ devices (RealTrack Systems, Almería, Spain) using Global Positioning System (GPS) technology sampling at 10 Hz. These devices utilize a dual-constellation configuration (Global Navigation Satellite System [GNSS] and GPS), which seems to provide more precision [24], and feature high-resolution triaxial accelerometers (1000 Hz), following standard protocol. For the analysis, data were collected in outdoor football fields, ensuring that no surrounding structures could interfere with the collection process. This equipment and its measurements are valid and reliable using the GNSS for time-motion analysis in football (distance covered variable: accuracy = 0.69–6.05%, test–retest reliability = 1.47, inter-unit reliability = 0.25; mean speed variable: accuracy = 0.18, intra-class correlation = 0.95, inter-unit reliability = 0.03), and have been awarded with the FIFA Quality Performance certificate [25,26].

The units were positioned between the shoulder blades, around the T3-T4 vertebral level, inside a dedicated pocket in a custom-fitted vest (dimensions of the devices = 81 × 45 × 16 mm), and were powered on 15 min before data acquisition, following manufacturer guidelines. To minimize variability between devices, each athlete consistently used the same WIMU^®^ unit for all sessions. The team regularly trained five 90 min training sessions per week, along with one competitive match each weekend.

All records were downloaded using the software SPRO 983 (RealTrack Systems, Almería, Spain) after the end of each session. Once the data were filtered through the software, they were imported into a Microsoft Excel spreadsheet (Microsoft Corporation, Redmond, WA, USA) to configure a matrix for subsequent analysis.

### 2.6. Statistical Analysis

The statistical analysis was conducted using the software Jamovi 2.6.2 [27,28] for Windows. Descriptive statistics data from the variables were presented using mean and standard deviation. A linear mixed model was carried out for each dependent variable in order to analyze the differences between the three WU protocols. Therefore, the “different WU protocols” was considered as fixed effect, while the “players’ ID” variable was considered as random effect. The Akaike information criterion (AIC) [29] and a likelihood ratio test [30] were used to select the model that best fitted each dependent variable. The restricted maximum likelihood (REML) estimation was used for model comparison with the same fixed effect’s structure but different random effects’ structures [30]. Significant results in the fixed effect on each variable were analyzed using post hoc pairwise comparison tests with Holm correction to assess differences between the levels. Marginal and conditional R^2^ metrics [31] were provided for each linear mixed model as a measure of effect sizes. Marginal R^2^ is concerned with variance explained by fixed effects, and conditional R^2^ is concerned with variance explained by both fixed and random effects [31]. The level of significance was set at *p* < 0.05.

## 3. Results

Table 1 shows the descriptive values (i.e., mean and standard deviation) of the duration and of the external and internal load variables for each of the three WU protocols.

Table 2 shows the results of the fixed effect omnibus tests, random components, and marginal and conditional R^2^ metrics on the dependent variables for each WU. The fixed effect “different WU protocols” showed statistically significant results (*p* < 0.05) in all variables, except in the variable nACC4 (*p* = 0.465). Marginal R^2^ values, reflecting variance explained by fixed effects alone, were low (0.00–0.16) for locomotor measures, indicating modest explanatory power of WU type. In contrast, higher conditional R^2^ values pointed to meaningful between-player variability, especially for decelerations and Player Load. Variables like duration and TRIMP Edwards showed high marginal and conditional R^2^ (>0.60), suggesting that WU protocol was a strong determinant of internal load and session volume, with minimal influence from individual differences.

Table 3 presents the results of the post hoc pairwise comparison tests for the fixed effect “different WU protocols” on the duration (in min) and the external and internal load variables.

Regarding the duration of the WUs, pre-match WU showed higher min than WU with ball (difference: 10.77; *p* < 0.001) and WU without ball (difference: 13.94; *p* < 0.001). WU with ball also showed higher values of min than WU without ball (difference: 3.17; *p* < 0.001).

In relation to the variables total distance, D0–45 and D45–60, WU without ball showed higher values than pre-match WU (difference: 25.76, 21.97 and 4.77, respectively; *p* < 0.001) and WU with ball (difference: 21.71, 15.44 and 4.72, respectively; *p* < 0.001). In the case of the variables D60–85 and D85–100, pre-match WU showed higher values than WU with ball (difference: 2.33 and 0.18, respectively; *p* < 0.001) and WU without ball (difference: 0.78 and 0.19, respectively; *p* = 0.021 and *p* < 0.001, respectively). WU without ball also showed higher values in the D60–85 than WU with ball (difference: 1.55; *p* < 0.001).

Regarding the maximum speed, higher values were observed for pre-match WU compared to WU with ball (difference: 5.49; *p* < 0.001) and WU without ball (difference: 5.16; *p* < 0.001). In the maximum acceleration, pre-match WU showed higher values than WU with ball (difference: 0.50; *p* < 0.001) and WU without ball (difference: 0.81; *p* < 0.001). WU with ball showed also higher values of maximum acceleration than WU without ball (difference: 0.30; *p* = 0.001). In the case of maximum deceleration, higher values were also observed for pre-match WU compared to WU with ball (difference: 0.93; *p* < 0.001) and WU without ball (difference: 1.06; *p* < 0.001).

Concerning the nACC1–2, pre-match WU showed higher values than WU with ball (difference: 0.56; *p* = 0.047). Significant differences were also found in the nACC2–3 and nACC3–4, where pre-match WU showed higher values than WU with ball (difference: 0.52 and 0.14, respectively; *p* < 0.001) and WU without ball (difference: 0.38 and 0.12, respectively; *p* < 0.001). WU without ball also showed a higher nACC2–3 than WU with ball (difference: 0.14; *p* = 0.021). In the case of the nDEC1–2, pre-match WU showed higher values than WU without ball (difference: 0.52; *p* = 0.011). Pre-match WU also showed higher values than WU with ball and WU without ball in the nDEC2–3 (difference: 0.52 and 0.31, respectively: *p* < 0.001), nDEC3–4 (difference: 0.20 and 0.16, respectively; *p* < 0.001), and nDEC4 (difference: 0.04 and 0.05, respectively; *p* < 0.001). WU without ball showed also higher nDEC2–3 and nDEC3–4 than WU with ball (difference: 0.20 and 0.04, respectively: *p* = 0.001 and *p* = 0.030, respectively).

Significant differences were also observed in the variable Player Load, where WU without ball showed higher values than WU with ball (difference: 0.28; *p* < 0.001) and pre-match WU (difference: 0.17; *p* = 0.001).

Finally, pre-match WU showed higher values in the variable TRIMPS Edwards than WU with ball (difference: 1.18; *p* < 0.001) and WU without ball (difference: 1.01; *p* < 0.001).

## 4. Discussion

The aim of the present study was to describe and compare the physical demands and physiological responses of various WU protocols in training sessions and official league matches in highly trained female football players. The results showed significant differences between WU protocols across most variables, suggesting that WU contents impact both the demands (external load) and responses (internal load), as well as activity duration. It is worth noting that the pre-match WU was the longest and most demanding protocol, with significantly higher values in variables such as distances covered at higher speeds, maximum speed, maximum acceleration, maximum deceleration, and number of accelerations and decelerations at high intensity.

Regarding the variable duration, the pre-match WU showed higher values than the two training WUs. This can be explained by reasons of design, structure, and context of the WU itself. Pre-match WUs are typically designed to comprehensively prepare the team for the immediate demands of competition. This implies a longer duration because it includes general physical components, specific technical–tactical tasks, progressive neuromuscular activations, and group dynamics that are part of the team’s psychological and cohesive routine before competing [1,2]. Unlike other, shorter and more specific types of WUs (e.g., WU with or without ball), the pre-match WU tends to be more comprehensive and, therefore, longer.

The pre-match WU is likely the most demanding protocol because this type of WUs is deliberately more intense, aiming to prepare players for the most demanding moment of the week (i.e., the competition) by enabling them to reach higher intensities and better cope with the subsequent physical demands. Additionally, although maximum efforts are rarely observed during WUs (as expected by their nature), high-intensity actions are still recommended, even if they are not often achieved. For instance, high-intensity WUs have been shown to more effectively enhance subsequent performance [10], provided their duration is limited to avoid excessive fatigue [32]. Despite the importance of including intense actions during WUs, it is also crucial to consider that, as the initial phase of a training session or match, players are typically exposed to high-intensity efforts progressively. This gradual buildup can influence the overall exposure to higher intensities. This was evident in the relatively short distances covered at speeds exceeding 85% of individual maximum speed across all WU protocols. Nevertheless, players did reach this threshold, and during the pre-match WU specifically, they covered approximately 3 m/min at speeds ranging from 60% to 85% of their maximum speed.

Differences in maximum values were markedly higher between the pre-match WU and the other two protocols (i.e., with ball and without ball). Regarding the maximum speed, the pre-match WU elicited higher values than the other two training WU protocols, a substantial difference that underscores the greater physical intensity and readiness promoted by the pre-match routine. Notably, these maximum speeds exceeded the thresholds commonly used to define very high-intensity running, and in the case of the pre-match WU, even surpassed typical sprint thresholds applied in female football populations [33], even though they remained lower than the maximum speeds usually achieved during competition [34]. Furthermore, maximum acceleration in the pre-match WU surpassed both training protocols, indicating substantially greater maximum effort. Interestingly, maximum accelerations achieved during the pre-match WU were similar to the magnitudes reported from a previous study that assessed linear sprints in youth (age: 17.4 ± 1.2 years) female football players [35]. In this regard, although the number of high-intensity accelerations (>3 m/s^2^) increased during the pre-match WU, their overall frequency remained low. To the best of our knowledge, this is the first study reporting maximum decelerations, which were also markedly higher in the pre-match WU than in the WUs with and without ball, as was the number of high-intensity decelerations (<−3 m/s^2^). However, given the low frequency of decelerations exceeding 3 m/s^2^ observed during competition [36], the maximum deceleration values reported in the present study seem comparable to the high-intensity efforts typically recorded in match play. These findings may reflect the intentional design of the pre-match WU to prepare players for peak match intensity, while athletes might delay reaching absolute maximum efforts until later phases of the session; differences may also be influenced by individual and sex-based capacity for maximum performance.

The variable TRIMP Edwards followed the same pattern, with the pre-match WU producing the highest values, reflecting the cumulative internal load differences between the protocols. This could be because pre-match WU reaches and maintains higher heart rate zones, more closely resembling the actual demands of the game. This pattern reflects the athletes’ physiological responses to the WUs, with the pre-match protocol imposing greater demands that could potentially contribute to higher fatigue; however, overall TRIMP values remain lower than those typically observed in small-sided games, such as 3 vs. 3 or 4 vs. 4 formats [37]. From a practical perspective, although higher-intensity pre-match WUs may better prepare players for the initial demands of competition, excessively demanding protocols could also potentially increase metabolic stress and contribute to residual fatigue if intensity and duration are not appropriately managed [2]. Therefore, practitioners should seek an appropriate balance between achieving sufficient activation and avoiding excessive pre-competition fatigue.

Beyond the differences observed between WU protocols, the marginal and conditional R^2^ values provide relevant information about the sources of variability in the players’ responses. The low marginal R^2^ values found for most locomotor variables indicate that WU type alone explained only a small proportion of the variance in these outcomes. Therefore, a substantial proportion of the variability in movement-related responses remained unexplained by WU type and may be attributable to other individual or contextual factors, such as player motivation, positional role, tactical involvement, movement strategy, neuromuscular profile, and fitness level. In contrast, the higher conditional R^2^ values observed for variables such as decelerations and Player Load suggest that between-player differences contributed meaningfully to the total explained variance. This may occur even within a highly trained sample, as decelerations and Player Load are particularly sensitive to repeated braking actions, changes in direction, multidirectional movement patterns, and individual movement strategies. Conversely, the high marginal and conditional R^2^ values observed for duration and TRIMP Edwards suggest that these outcomes were more directly determined by the structure and duration of the WU protocol itself. From an applied perspective, these findings reinforce the need to interpret WU responses not only at the group level, but also through an individualized monitoring approach.

This study is not exempt from limitations. The sample, in addition to being small, was specific to a particular context and population, which may limit the generalizability of the findings. Furthermore, as the data were collected from a single team, the results may have been influenced by that team’s specific tactical philosophy and playing style. In addition, playing position was not considered in the analyses, despite the possibility that the physical and tactical requirements of the pre-match WU may differ according to positional roles. Therefore, future studies should include players from multiple teams and account for positional specificity when analyzing WU demands. Another limitation of the study is that the WU protocols were predefined, whereas in practice, coaches may select from a broader range of exercises, each potentially impacting players differently. Moreover, although the present study analyzed and compared the demands associated with different WU protocols, their subsequent effects on football-specific performance were not examined. Future research should also investigate whether these different WU strategies influence subsequent physical and physiological performance during training or competition. Additionally, although running speeds were normalized to individual maximum values, accelerations and decelerations were based on commonly used but ultimately arbitrary thresholds. Finally, the menstrual cycle phase was not controlled in the present study, which should be acknowledged as a limitation given that hormonal fluctuations may influence individual physiological and perceptual responses during exercise.

## 5. Conclusions

This study highlights that different WU designs are associated with distinct physical demands and physiological responses in elite female football players. The pre-match WU consistently imposed the highest external and internal loads, emphasizing its role in priming players for competition. Differences in maximum values, particularly for speed, acceleration and deceleration, underscore the heightened intensity and readiness elicited by this protocol. These findings stress the importance of tailoring WU content to the desired outcomes of the training session or match, especially when preparing athletes for high-intensity performance. Moreover, they contribute to female-specific research, helping practitioners develop WU strategies that support both efficient performance and effective fatigue management.

## Figures and Tables

**Table 1:** Mean and standard deviation (SD) of the measured variables for each warm-up (WU) protocol.

Variables	WU with Ball		WU without Ball		Pre-Match WU	
	Mean	SD	Mean	SD	Mean	SD
Duration (min)	9.73	2.57	6.57	2.55	20.51	2.05
Total distance (m/min)	68.86	18.21	90.57	31.53	64.80	14.23
D0–45 (m/min)	64.75	16.18	80.19	26.29	58.22	12.06
D45–60 (m/min)	3.44	2.85	8.16	11.74	3.39	1.65
D60–85 (m/min)	0.64	0.78	2.19	2.99	2.97	1.27
D85–100 (m/min)	0.03	0.11	0.02	0.25	0.21	0.47
Maximum speed (km/h)	17.74	3.49	18.12	2.95	23.20	3.14
Maximum acceleration (m/s^2^)	3.37	0.89	3.07	0.71	3.87	0.62
Maximum deceleration (m/s^2^)	−3.15	0.69	−3.06	0.68	−4.09	0.84
nACC1–2 (/min)	3.68	1.54	3.85	1.49	4.22	0.91
nACC2–3 (/min)	0.64	0.55	0.78	0.46	1.16	0.47
nACC3–4 (/min)	0.09	0.25	0.12	0.18	0.23	0.14
nACC4 (/min)	0.03	0.05	0.02	0.08	0.03	0.05
nDEC1–2 (/min)	3.65	1.52	3.53	1.30	4.00	0.98
nDEC2–3 (/min)	0.62	0.56	0.84	0.53	1.13	0.36
nDEC3–4 (/min)	0.08	0.11	0.13	0.16	0.28	0.17
nDEC4 (/min)	0.02	0.04	0.01	0.05	0.06	0.08
Player Load (AU/min)	0.97	0.25	1.26	0.44	1.05	0.23
TRIMPS Edwards (AU/min)	1.39	0.92	1.56	0.99	2.54	1.05

Note: D0–45 is the distance covered within 0–45% of individual maximum speed, D45–60 is the distance covered within 45–60% of individual maximum speed, D60–85 is the distance covered within 60–85% of individual maximum speed, D85–100 is the distance covered within 85–100% of individual maximum speed, nACC1–2 is the number of accelerations of magnitudes between 1 and 2 m/s^2^, nACC2–3 is the number of accelerations of magnitudes between 2 and 3 m/s^2^, nACC3–4 is the number of accelerations of magnitudes between 3 and 4 m/s^2^, nACC4 is the number of accelerations of magnitudes above 4 m/s^2^, nDEC1–2 is the number of decelerations of magnitudes between −1 and −2 m/s^2^, nDEC2–3 is the number of decelerations of magnitudes between −2 and −3 m/s^2^, nDEC3–4 is the number of decelerations of magnitudes between −3 and −4 m/s^2^, nDEC4 is the number of decelerations of magnitudes below −4 m/s^2^, and AU is arbitrary units.

**Table 2:** Results of the linear mixed models in the fixed effect different warm-up (WU) protocols on the measured variables.

Variables	Fixed Effect Omnibus Tests		Random Components				Marginal R^2^/Conditional R^2^
	F	*p*	Player ID		Residual		
			Variance	SD	Variance	SD	
Duration (min)	841.16	<0.001	0.00	0.00	6.14	2.48	0.81/0.81
Total distance (m/min)	36.77	<0.001	0.00	0.00	717.41	26.78	0.16/0.16
D0–45 (m/min)	32.90	<0.001	0.15	0.39	506.21	22.50	0.14/0.14
D45–60(m/min)	12.19	<0.001	0.00	0.00	87.03	9.33	0.06/0.06
D60–85 (m/min)	19.61	<0.001	0.00	0.00	5.91	2.43	0.09/0.09
D85–100 (m/min)	12.90	<0.001	0.00	0.05	0.08	0.28	0.06/0.09
Maximum speed (km/h)	83.38	<0.001	0.66	0.81	9.00	3.00	0.29/0.34
Maximum acceleration (m/s^2^)	32.47	<0.001	0.00	0.02	0.55	0.74	0.14/0.14
Maximum deceleration (m/s^2^)	62.58	<0.001	0.04	0.21	0.47	0.68	0.23/0.30
nACC1–2 (/min)	3.19	0.042	0.07	0.27	1.94	1.39	0.02/0.05
nACC2–3 (/min)	23.37	<0.001	0.00	0.00	0.23	0.48	0.11/0.11
nACC3–4 (/min)	12.56	<0.001	0.00	0.04	0.04	0.19	0.06/0.09
nACC4 (/min)	0.77	0.465	0.00	0.01	0.01	0.07	0.00/0.01
nDEC1–2 (/min)	4.31	0.014	0.10	0.31	1.61	1.27	0.02/0.08
nDEC2–3 (/min)	19.61	<0.001	0.01	0.08	0.26	0.51	0.09/0.11
nDEC3–4 (/min)	41.92	<0.001	0.00	0.05	0.02	0.14	0.16/0.27
nDEC4 (/min)	24.73	<0.001	0.00	0.00	0.00	0.05	0.11/0.11
Player Load (AU/min)	21.07	<0.001	0.02	0.13	0.13	0.36	0.09/0.20
TRIMPS Edwards (AU/min)	52.31	<0.001	0.40	0.63	0.58	0.76	0.14/0.49

Note: D0–45 is the distance covered within 0–45% of individual maximum speed, D45–60 is the distance covered within 45–60% of individual maximum speed, D60–85 is the distance covered within 60–85% of individual maximum speed, D85–100 is the distance covered within 85–100% of individual maximum speed, nACC1–2 is the number of accelerations of magnitudes between 1 and 2 m/s^2^, nACC2–3 is the number of accelerations of magnitudes between 2 and 3 m/s^2^, nACC3–4 is the number of accelerations of magnitudes between 3 and 4 m/s^2^, nACC4 is the number of accelerations of magnitudes above 4 m/s^2^, nDEC1–2 is the number of decelerations of magnitudes between −1 and −2 m/s^2^, nDEC2–3 is the number of decelerations of magnitudes between −2 and −3 m/s^2^, nDEC3–4 is the number of decelerations of magnitudes between −3 and −4 m/s^2^, nDEC4 is the number of decelerations of magnitudes below −4 m/s^2^, and AU is arbitrary units. Significance level set at *p* < 0.05.

**Table 3:** Post hoc tests results for the fixed effect different warm-up (WU) protocols on the measured variables.

Variables	Comparison			Difference	SE	t	df	p^holm^
	WU	vs.	WU					
Duration (min)	WU with ball	-	WU without ball	3.17	0.31	10.17	389.78	<0.001
	WU with ball	-	Pre-match WU	−10.77	0.40	−26.64	394.98	<0.001
	WU without ball	-	Pre-match WU	−13.94	0.34	−40.74	392.80	<0.001
Total distance (m/min)	WU with ball	-	WU without ball	−21.71	3.37	−6.45	389.78	<0.001
	WU with ball	-	Pre-match WU	4.06	4.37	0.93	394.98	0.354
	WU without ball	-	Pre-match WU	25.76	3.70	6.97	392.80	<0.001
D0–45 (m/min)	WU with ball	-	WU without ball	−15.44	2.83	−5.46	389.74	<0.001
	WU with ball	-	Pre-match WU	6.52	3.67	1.78	394.99	0.076
	WU without ball	-	Pre-match WU	21.97	3.11	7.07	392.89	<0.001
D45–60 (m/min)	WU with ball	-	WU without ball	−4.72	1.17	−4.02	389.78	<0.001
	WU with ball	-	Pre-match WU	0.05	1.52	0.03	394.98	0.974
	WU without ball	-	Pre-match WU	4.77	1.29	3.70	392.80	<0.001
D60–85 (m/min)	WU with ball	-	WU without ball	−1.55	0.31	−5.07	389.78	<0.001
	WU with ball	-	Pre-match WU	−2.33	0.40	−5.87	394.98	<0.001
	WU without ball	-	Pre-match WU	−0.78	0.34	−2.33	392.80	0.021
D85–100 (m/min)	WU with ball	-	WU without ball	0.01	0.04	0.25	386.89	0.806
	WU with ball	-	Pre-match WU	−0.18	0.05	−4.02	392.60	<0.001
	WU without ball	-	Pre-match WU	−0.19	0.04	−4.96	394.77	<0.001
Maximum speed (km/h)	WU with ball	-	WU without ball	−0.33	0.38	−0.86	383.91	0.388
	WU with ball	-	Pre-match WU	−5.49	0.50	−11.09	387.81	<0.001
	WU without ball	-	Pre-match WU	−5.16	0.42	−12.27	391.38	<0.001
Maximum acceleration (m/s^2^)	WU with ball	-	WU without ball	0.30	0.09	3.25	389.73	0.001
	WU with ball	-	Pre-match WU	−0.50	0.12	−4.17	394.99	<0.001
	WU without ball	-	Pre-match WU	−0.81	0.10	−7.88	392.93	<0.001
Maximum deceleration (m/s^2^)	WU with ball	-	WU without ball	0.13	0.09	1.49	382.99	0.138
	WU with ball	-	Pre-match WU	−0.93	0.11	−8.29	386.28	<0.001
	WU without ball	-	Pre-match WU	−1.06	0.10	−11.09	389.89	<0.001
nACC1–2 (/min)	WU with ball	-	WU without ball	−0.15	0.18	−0.84	386.06	0.402
	WU with ball	-	Pre-match WU	−0.56	0.23	−2.43	391.35	0.047
	WU without ball	-	Pre-match WU	−0.41	0.19	−2.10	394.16	0.072
nACC2–3 (/min)	WU with ball	-	WU without ball	−0.14	0.06	−2.31	389.78	0.021
	WU with ball	-	Pre-match WU	−0.52	0.08	−6.57	394.98	<0.001
	WU without ball	-	Pre-match WU	−0.38	0.07	−5.66	392.80	<0.001
nACC3–4 (/min)	WU with ball	-	WU without ball	−0.03	0.02	−1.24	385.98	0.216
	WU with ball	-	Pre-match WU	−0.14	0.03	−4.69	391.23	<0.001
	WU without ball	-	Pre-match WU	−0.12	0.03	−4.40	394.08	<0.001
nACC4 (/min)	WU with ball	-	WU without ball	0.01	0.01	0.78	389.07	0.874
	WU with ball	-	Pre-match WU	−0.00	0.01	−0.34	394.87	0.874
	WU without ball	-	Pre-match WU	−0.01	0.01	−1.11	394.14	0.798
nDEC1–2 (/min)	WU with ball	-	WU without ball	0.14	0.16	0.85	384.53	0.397
	WU with ball	-	Pre-match WU	−0.38	0.21	−1.83	388.84	0.136
	WU without ball	-	Pre-match WU	−0.52	0.18	−2.92	392.30	0.011
nDEC2–3 (/min)	WU with ball	-	WU without ball	−0.20	0.06	−3.20	386.88	0.001
	WU with ball	-	Pre-match WU	−0.52	0.08	−6.23	392.58	<0.001
	WU without ball	-	Pre-match WU	−0.31	0.07	−4.44	394.77	<0.001
nDEC3–4 (/min)	WU with ball	-	WU without ball	−0.04	0.02	−2.18	381.29	0.030
	WU with ball	-	Pre-match WU	−0.20	0.02	−8.56	383.54	<0.001
	WU without ball	-	Pre-match WU	−0.16	0.02	−8.10	386.87	<0.001
nDEC4 (/min)	WU with ball	-	WU without ball	0.01	0.01	1.00	389.37	0.320
	WU with ball	-	Pre-match WU	−0.04	0.01	−5.12	394.97	<0.001
	WU without ball	-	Pre-match WU	−0.05	0.01	−6.96	393.66	<0.001
Player Load (AU/min)	WU with ball	-	WU without ball	−0.28	0.05	−6.17	381.39	<0.001
	WU with ball	-	Pre-match WU	−0.11	0.06	−1.84	383.71	0.067
	WU without ball	-	Pre-match WU	0.17	0.05	3.40	387.06	0.001
TRIMPS Edwards (AU/min)	WU with ball	-	WU without ball	−0.17	0.10	−1.74	376.55	0.083
	WU with ball	-	Pre-match WU	−1.18	0.13	−9.32	376.94	<0.001
	WU without ball	-	Pre-match WU	−1.01	0.11	−9.36	378.21	<0.001

Note: D0–45 is the distance covered within 0–45% of individual maximum speed, D45–60 is the distance covered within 45–60% of individual maximum speed, D60–85 is the distance covered within 60–85% of individual maximum speed, D85–100 is the distance covered within 85–100% of individual maximum speed, nACC1–2 is the number of accelerations of magnitudes between 1 and 2 m/s^2^, nACC2–3 is the number of accelerations of magnitudes between 2 and 3 m/s^2^, nACC3–4 is the number of accelerations of magnitudes between 3 and 4 m/s^2^, nACC4 is the number of accelerations of magnitudes above 4 m/s^2^, nDEC1–2 is the number of decelerations of magnitudes between −1 and −2 m/s^2^, nDEC2–3 is the number of decelerations of magnitudes between −2 and −3 m/s^2^, nDEC3–4 is the number of decelerations of magnitudes between −3 and −4 m/s^2^, nDEC4 is the number of decelerations of magnitudes below −4 m/s^2^, and AU is arbitrary units, SE is Standard Error, and df is degrees of freedom. Significance level set at *p* < 0.05.

## Data Availability

Data are contained within the article.
